# Reduction of the HIV Protease Inhibitor-Induced ER Stress and Inflammatory Response by Raltegravir in Macrophages

**DOI:** 10.1371/journal.pone.0090856

**Published:** 2014-03-13

**Authors:** Xiaoxuan Zhang, Risheng Cao, Runping Liu, Renping Zhao, Yi Huang, Emily C. Gurley, Phillip B. Hylemon, William M. Pandak, Guangji Wang, Luyong Zhang, Xiaokun Li, Huiping Zhou

**Affiliations:** 1 Center of Drug Metabolism and Pharmacokinetics, China Pharmaceutical University, Nanjing, P.R.China; 2 Department of Microbiology & Immunology, Virginia Commonwealth University, Richmond, Virginia, United States of America; 3 School of Pharmacy, Wenzhou Medical University, Wenzhou, P.R.China; 4 Department of Internal Medicine/Gastroenterology and McGuire Veterans Affairs Medical Center, Richmond, Virginia, United States of America; Northeast Ohio Medical University, United States of America

## Abstract

**Background:**

HIV protease inhibitor (PI), the core component of highly active antiretroviral treatment (HAART) for HIV infection, has been implicated in HAART-associated cardiovascular complications. Our previous studies have demonstrated that activation of endoplasmic reticulum (ER) stress is linked to HIV PI-induced inflammation and foam cell formation in macrophages. Raltegravir is a first-in-its-class HIV integrase inhibitor, the newest class of anti-HIV agents. We have recently reported that raltegravir has less hepatic toxicity and could prevent HIV PI-induced dysregulation of hepatic lipid metabolism by inhibiting ER stress. However, little information is available as to whether raltegravir would also prevent HIV PI-induced inflammatory response and foam cell formation in macrophages.

**Methodology and Principal Findings:**

In this study, we examined the effect of raltegravir on ER stress activation and lipid accumulation in cultured mouse macrophages (J774A.1), primary mouse macrophages, and human THP-1-derived macrophages, and further determined whether the combination of raltegravir with existing HIV PIs would potentially exacerbate or prevent the previously observed activation of inflammatory response and foam cell formation. The results indicated that raltegravir did not induce ER stress and inflammatory response in macrophages. Even more interestingly, HIV PI-induced ER stress, oxidative stress, inflammatory response and foam cell formation were significantly reduced by raltegravir. High performance liquid chromatography (HPLC) analysis further demonstrated that raltegravir did not affect the uptake of HIV PIs in macrophages.

**Conclusion and Significance:**

Raltegravir could prevent HIV PI-induced inflammatory response and foam cell formation by inhibiting ER stress. These results suggest that incorporation of this HIV integrase inhibitor may reduce the cardiovascular complications associated with current HAART.

## Introduction

The development of HIV protease inhibitors (HIV PIs) is one of the most significant advances of the past two decades in controlling HIV infection. Incorporation of HIV PIs in HAART has had a profound impact on the natural history of HIV and AIDS. However, in the era of HAART, drug-induced metabolic toxicity has emerged as an important complication of combination antiretroviral therapy, particularly of those regimens containing HIV PIs [1]–[3]. HIV PI-induced dyslipidemia and inflammation are two major risk factors of cardiovascular complications in HIV patients under HAART.

Previous studies from other laboratories and ours suggest that HIV PI-induced endoplasmic reticulum (ER) stress response and subsequent activation of the unfolded protein response (UPR) represent an important cellular signaling mechanism of HIV PI-induced metabolic syndromes (dyslipidemia, insulin-resistance, lipodystrophy/lipoatrophy) [1], [2], [4]–[9]. ER stress has been closely linked to various human diseases including inflammatory diseases, cardiovascular diseases, diabetes, and all types of human liver diseases [10]. Our previous *in vitro* studies have demonstrated that most HIV PIs not only induce the accumulation of intracellular free cholesterol and lipid and activate the UPR in hepatocytes and macrophages, but they also increase the release of inflammatory cytokines, promote foam cell formation and induce cell apoptosis in macrophages [5], [6].

HIV integrase inhibitor is a new class of antiviral agents used to treat HIV-1-infected patients. Raltegravir (also known as MK0518, Isentress) is a first-in-its-class oral integrase inhibitor and has demonstrated potent efficacy against multidrug-resistant HIV-1 and was initially approved by the FDA in 2007 to treat treatment-experienced HIV-1-infected patients [11]. Recently, raltegravir in combination treatment was listed as one of the preferred regimens recommended for treatment-naive HIV-1-infected patients. Clinical studies have shown that raltegravir was well-tolerated and had fewer side effects compared to other classes of antiretroviral drugs, such as HIV PIs and reverse transcriptase inhibitors [12]–[14]. Our recent study demonstrated that raltegravir is able to prevent HIV PI-induced dysregulation of hepatic lipid metabolism by inhibiting the ER stress response [15]. However, little information is available as to whether raltegravir would have a similar protective effect against HIV PI-induced inflammatory response and dysregulation of lipid metabolism in macrophages. The current study was aimed at examining the effect of raltegravir on HIV PI-induced inflammatory response, foam cell formation and dysregulation of lipid metabolism in macrophages, and further determining whether the combination of this integrase inhibitor with existing, most commonly used HIV PIs (lopinavir and ritonavir) could potentially prevent the previously observed development of inflammatory response and foam cell formation.

## Methods

### Materials

Antibodies against C/EBP homologous protein (CHOP), activating transcription factor-4 (ATF-4), X-box-binding protein-1 (XBP-1), lamin B, horseradish peroxidase (HRP)-conjugated donkey anti-goat IgG and HRP-conjugated goat anti-rabbit IgG were from Santa Cruz Biotechnology (Santa Cruz, CA). Mouse monoclonal antibody against β-actin was from Calbiochem (San Diego, CA). Mouse monoclonal antibody against CD36 was from Cayman Chemical (Ann Arbor, MI). Rabbit polyclonal antibody against scavenger receptor A (SRA) was from R&D Systems (Minneapolis, MN). Acetylated-LDL and oxidized-LDL were from Intracel (Frederick, MD). Carboxy-DCFDA and carboxy-H_2_DCFDA were from Invitrogen (Grand Island, NY). Bio-Rad protein assay reagent, Criterion XT Precast Gel and Precision Plus Protein Kaleidoscope Standards were obtained from Bio-Rad (Hercules, CA). Kits for total cholesterol, free cholesterol, and triglyceride were from Wako (Richmond, VA). Total RNA isolation kit was from Promega (Madison, WI). High-capacity cDNA archive kit was from Applied Biosystems (Foster City, CA). All other chemical reagents were from Sigma (St. Louis, MO).

### Cell Culture

Mouse J774A.1 macrophages (ATCC, Rockville MD, USA) were maintained in DMEM supplemented with 10% FBS, 100 U/ml penicillin, and 100 µg/ml streptomycin at 37°C with 5% CO_2_. Cells from passages six to nine were used in these studies. Human THP-1 monotypic cells (ATCC, Rockville MD, USA) were cultured in RPMI 1640 medium supplemented with 10% FBS, 100 U/ml penicillin, and 100 µg/ml streptomycin at 37°C with 5% CO_2_. THP-1 monocytes were treated with PMA (100 ng/ml) for 5 days to facilitate differentiation into macrophages. HIV PIs (ritonavir and lopinavir) and Raltegravir were dissolved in dimethyl sulfoxide (DMSO) and added directly to culture medium (final concentrations 5 to 25 µM) and incubated for 0.5 to 24 h. For each result, a minimum of three independent experiments was performed. Cell viability was assessed with the Cellometer Vision CBA Analysis System (Nexcelom Bioscience, Lawrence, MA) and with the use of trypan blue.

### Isolation and Culture of Primary Mouse Kupffer Cells and Mouse Peritoneal Macrophages

Primary mouse Kupffer cells were isolated from male C57BL/6 mice (male 8-week old) using a Percoll gradient centrifugation method as described previously by Jacob, et al [16]. Isolated mouse Kupffer cells were cultured in DMEM supplemented with 10% FBS, 20% L-cell media, 100 U/ml penicillin, and 100 µg/ml streptomycin at 37°C with 5% CO_2_. In order to isolate primary mouse peritoneal macrophages, adult male C57BL/6 mice were injected intraperitoneally with 0.5 ml of phosphate-buffered saline (PBS) containing 40 µg of concanavalin A. The macrophages were harvested 72 h after injection by peritoneal lavage. The harvested cells were cultured in DMEM containing 10% fetal bovine serum (FBS) and 20% L-cell-conditioned medium [6]. The medium was replaced every 24 h until the macrophages were confluent. Animal studies were approved by Institutional Animal Care and Use Committee of Virginia Commonwealth University and were conducted in accordance with the Declaration of Helsinki, the Guide for the Care and Use of Animals (National Academy Press, Washington, D.C., 1996), and all applicable regulations.

### High-performance Liquid Chromatography (HPLC) Analysis of Intracellular Uptake of HIV PIs and Raltegravir in Macrophages

Mouse J774A.1 macrophages were treated with HIV PIs and raltegravir (0 to 25 µM) for various time periods (0 to 24 h). The culture medium and total cell lysates of each time point were collected. The drugs in media and cells were extracted using solid phase C-18 cartridges as described previously [5]. An Agilent 1200 Series HPLC system and a ZORBAX eclipse C18 reverse phase column (5 µm, 4.6 mm×25 cm, Agilent Technologies, Santa Clara, CA) were used to quantify the HIV PIs and raltegravir in cells. The gradient mobile phase for raltegravir consisted of acetonitrile (A) and 0.01% triethylamine (B) adjusted to pH 3.0 by phosphoric acid. The starting mobile phase consisted of 40% A and 60% B (vol/vol) and was switched to 70% A and 30% B at 4 min, then changed back to 40% A and 60% B (v/v) at 6 min. The mobile phase was delivered at 1 ml/min. The fluorescence detector was used to detect raltegravir using excitation/emission wavelengths of 299/396 nm [17]. The mobile phase for ritonavir and lopinavir was acetonitrile: 20 mM sodium dihydrogenphosphate, pH 6 [60∶40(v/v)] and 0.025% triethylamine. The mobile phase was delivered at 1 ml/min. The peaks of ritonavir and lopinavir were detected at 210 nm. A standard curve of each drug was constructed using weighted linear regression of peak area ratio values of the calibration standards. The percentage of drug recovery after the solid-phase extraction was determined by comparing the extracted internal standard.

### Enzyme-linked Immunosorbent Assays (ELISA) of Cytokines

Mouse J774A.1 macrophages were treated with HIV PIs with or without raltegravir for 24 h. At the end of the treatment, the culture media were collected and centrifuged at 14,000×rpm for 5 min. The supernatants were stored in aliquots at −70°C. TNF-α and IL-6 levels in the media were determined by ELISA using mouse TNF-α and mouse IL-6 ELISA Max™ Set Deluxe Kits (BioLegend). The total protein concentrations of the viable cell pellets were determined using Bio-Rad Protein Assay reagent. Total amounts of the TNF-α and IL-6 in media were normalized to the total protein amounts of the viable cell pellets.

### Analysis of Apoptosis by Annexin V and Propidium Iodide Staining

Mouse J774A.1 cells or human THP-1-derived macrophages were treated with HIV PIs with or without raltegravir (25 µM) for 24 h and stained with Annexin V-FITC and propidium iodide using BD ApoAlert Annexin V kit, according to the protocol recommended by the manufacturer. Annexin V/propidium iodide-stained cells were visualized under fluorescence microscopy with a 40×objective using a dual-filter set for FITC and rhodamine [5], [6]. The relative fluorescence density was determined using IPLab 4.0 software.

### Western Blot Analysis

The nuclear and cytosolic proteins were prepared as previously described [5]. The protein concentration was determined using the Bio-Rad protein assay reagent. The nuclear extracts (30 µg of protein) or cytosol extracts (70 µg of protein) or total cell lysate proteins (80 µg) were resolved on 10% or 7% or 12% Bis-Tris NuPAGE Novex gels or 10% Criterion XT precast gels and transferred to nitrocellulose membranes. Immunoblots were blocked overnight at 4°C with 5% non-fat milk in TBS buffer and incubated with primary antibodies. Immunoreactive bands were detected using horseradish peroxidase–conjugated secondary antibody and the Western Lightning Chemiluminescence Reagent Plus. The density of the immunoblot bands was analyzed using Imagelab software (Biorad, CA).

### Real-time Quantitative PCR

Total cellular RNA was isolated after treatment with HIV PIs and raltegravir, or vehicle control (DMSO) for 24 h, using the Promega SV Total RNA Isolation System. Total RNA (2 µg) was used for the first-strand cDNA synthesis using the High-Capacity cDNA Archive Kit. The mRNA levels of CHOP, spliced form of XBP-1 (sXBP-1), ATF-4, CD36, scavenger receptor A (SRA), ATP-binding cassette transporter A1 (ABCA1), and ABCG1 were quantified using specific primers for each gene (Table 1). iQ™ SYBR Green Supermix (Bio-Rad Laboratories) was used as a fluorescent dye to detect the presence of double-stranded DNA. The mRNA values for each gene were normalized to internal control β-actin mRNA. The ratio of normalized mean value for each treatment group to vehicle control group was calculated.

**Table 1:** Real time PCR primers.

Gene	Species	Gene bank Accession No	Forward Primer 5′ to 3′	Reverse Primer 5′ to 3′
ABCA1	Human	NM_005502	AGGCTGTGTCTCGTATTGTC	GAGTTGTAGAGTTGTCATAGAAGG
ABCG1	Human	NM_004915	CCGACCGACGACACAGAGA	GCACGAGACACCCACAAACC
ATF-4	Human	NM_001675	CAACAACAGCAAGGAGGATG	AATTGGGTTCACCGTCTGG
CD36	Human	NM_001001548	TGTCATTGGTGCTGTCCTG	TGTTGCTGCTGTTCATCATC
CHOP	Human	S40706	CTGAATCTGCACCAAGCATGA	AAGGTGGGTAGTGTGGCCC
IL-6	Human	NM_000600	CAGATTTGAGAGTAGTGAGGAAC	CGCAGAATGAGATGAGTTGTC
SRA	Human	NM_138715	TTCACAATCAACAGGAGGACAC	GCAAACACGAGGAGGTAAAGG
TNF-α	Human	NM_000594	CGAGTCTGGGCAGGTCTAC	GGGAGGCGTTTGGGAAGG
XBP-1s	Human	NM_005080	TCCGCAGCACTCAGACTAC	TCCAAGTTGTCCAGAATGCC

### Oil Red O and Nile Red Staining

Cells were treated with HIV PIs with or without raltegravir for 24 h. Cells were fixed in 3.7% formaldehyde for 30 min. After washing with PBS 3 times, the intracellular lipid was stained with Oil Red O or Nile Red as described previously [6], [18]. The images were taken under 40×lenses using an Olympus microscope equipped with an image recorder. Images of Nile-Red staining were obtained under a 40×objective using a FITC filter on a fluorescent microscope (Olympus, Center Valley, PA).

### Measurement of Intracellular Free Cholesterol, Total Cholesterol and Triglyceride

Mouse J774A.1 macrophages were plated on 60-mm plates overnight and then treated with control vehicle or HIV PIs for 24 h. At the end of the treatment, the cells were washed with PBS twice. The intracellular levels of total cholesterol, free cholesterol, triglyceride, were measured using free cholesterol, cholesterol E and triglyceride assay kits (Wako Bioproducts, Richmond, VA) and normalized by total protein amount as described previously [15].

### Assay of Endoplasmic Reticulum Calcium Pools

Mouse J774A.1 macrophages were grown on 22×40-mm coverslips and treated with vehicle or ritonavir with or without raltegravir for 24 h. The cells were loaded with 4 µM Fura-2 AM and 0.3% Plurinic F-127 in HBSS at 37°C for 60 min and incubated in HBSS for an additional 30 min, then mounted on the stage of an Axioskop 2 plus upright fluorescence microscope (Carl Zeiss GmbH, Jena, Germany) equipped with a 40×objective. After washing with HBSS without Ca^2+^ and Mg^2+^ three times, the cells were stimulated with 100 nM thapsigargin (TG) to induce the calcium release from ER. Fluorescence images (510-nm emission after alternate 340- and 380-nm excitation) before and after addition of TG were collected at 15-ms intervals through a cooled charge-coupled device camera (TILL Photonics LLC, Martinsreid, Germany) which is attached to an image intensifier (VS4-1845; Videoscope, Washington, DC), an epifluorescent light source (Polychrome IV; TILL Photonics), a 515 nm dichroic beam splitter, and a 535 nm emission filter (20 nm band pass; Omega Optical, Brattleboro, VT). The 340∶380 ratios of individual cells in these images were analyzed using TILLvisION version 3.1 imaging software [6].

### Measurement of Reactive Oxygen Species (ROS) by Flow Cytometry

ROS production was detected in mouse J774A.1 cells loaded with C-H_2_DCFDA-AM (Invitrogen), which was hydrolyzed by intracellular esterases and oxidized to fluorescent carboxy-DCF (excitation 488 nm; emission 525 nm), primarily by H_2_O_2_ in the presence of peroxidase. Cells were treated with HIV PI with or without raltegravir for 24 h, and then incubated with C-H_2_DCFDA-AM (5 µM) for 30 min (37°C). Cells without dye were used as negative control and H_2_O_2_ treated cells were used as a positive control. Cells were collected and washed with PBS. Cells were analyzed by flow cytometry using a Beckman Coulter (EPICS XL). In order to determine the effect of HIV PIs and raltegravir on dye uptake in macrophages, J774A.1 cells were loaded with the cell-permanent, oxidized form of the dye (DCFDA-AM, 5 µM) and treated with HIV PIs with or without raltegravir for 4 h. The fluorescence intensity was measured using a 96-well plate reader.

### Statistical Analysis

All results were expressed as mean ± standard error of the mean. One-way ANOVA and Student’s t test were used to analyze the differences between different treatments. Statistics were performed using GraphPad Pro. A probability (p) of less than 0.05 was considered statistically significant.

## Results

### Uptake of Raltegravir in Macrophages

**Figure 1:**
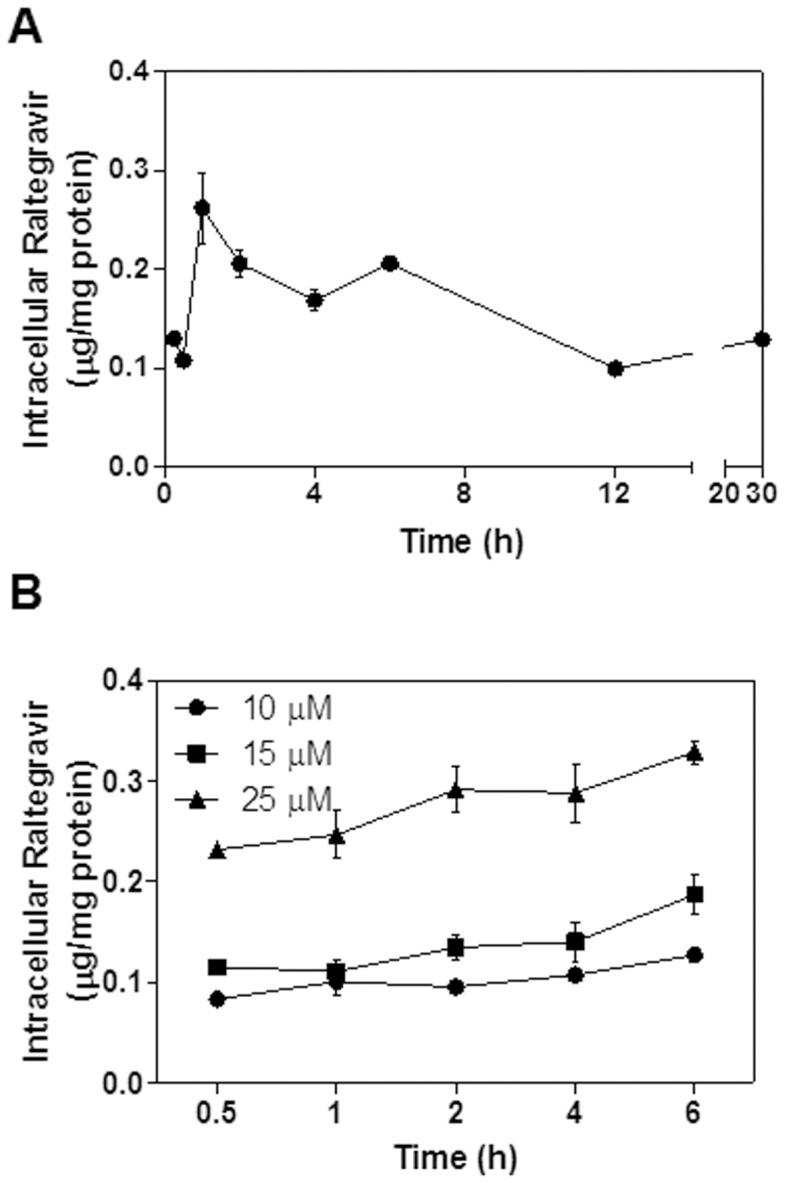
Time and dose-dependent uptake of raltegravir in macrophages. (**A**). Mouse J774A.1 cells were treated with raltegravir (25 µM) for various time periods (0.5, 1, 2, 3, 4 6, 12, and 24 h). At the end of each treatment period, the cells were collected and intracellular drug concentration was determined by HPLC and normalized with total cellular protein amount as described in “Methods”. The graphic representation of intracellular drug levels over time is shown. (**B**). Mouse J774A.1 cells were treated with different concentrations of raltegravir (10, 15, and 25 µM) for various time periods (0.5, 1, 2, 4, and 6 h). The intracellular drug concentration was determined as described above.

In order to study the effect of raltegravir on HIV PI-induced inflammation and dysregulation of lipid metabolism in macrophages, we first examined the uptake of raltegravir in macrophages. Mouse J774A.1 cells were incubated with raltegravir (25 µM) for different time periods (0.5, 1, 2, 3, 4, 6, 12, and 24 h) or with different amounts of raltegravir (10, 15, or 25 µM) for 0.5, 1, 2, 4 or 6 h. The intracellular drug concentrations of raltegravir were determined by HPLC analysis. The results indicated that raltegravir was rapidly and dose-dependently taken up by macrophages (Fig. 1).

### Effect of Raltegravir on HIV PI-induced UPR Activation and Apoptosis

**Figure 2:**
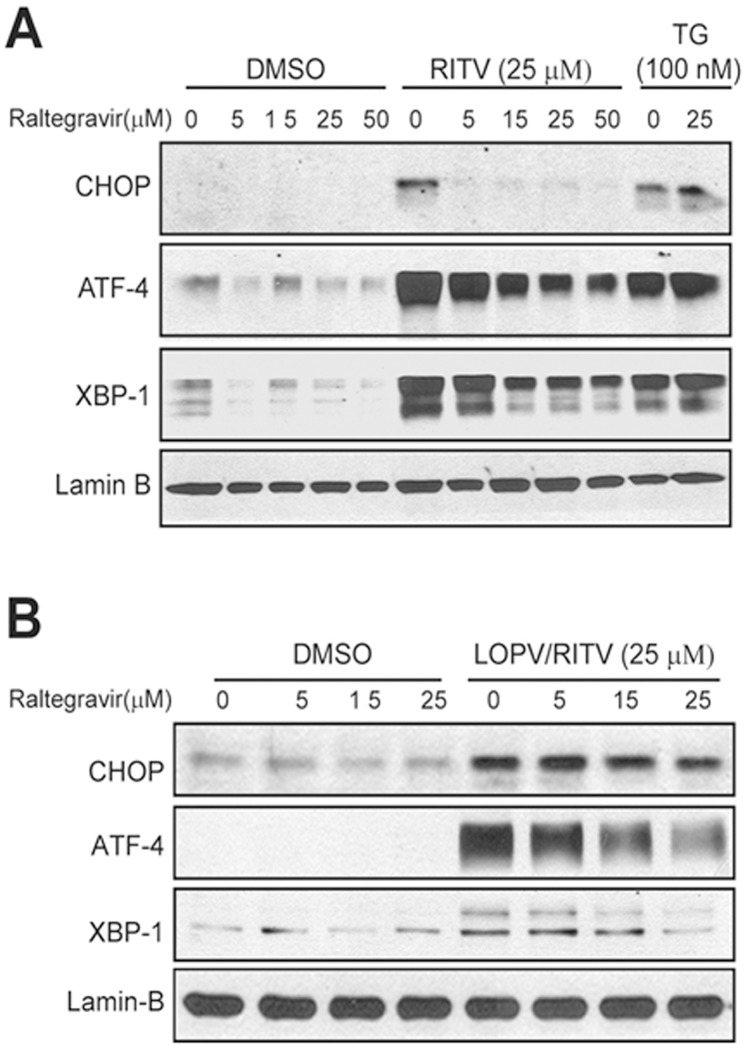
Effect of raltegravir on HIV PI-induced UPR activation in macrophages. (**A**). Mouse J774A.1 cells were treated with different concentrations of raltegravir (0, 5, 15, 25 or 50 µM) in the absence or presence of ritonavir (RITV, 25 µM) or thapsigargin (TG, 100 nM), a known ER stress inducer, for 4 h, and the nuclear proteins were isolated. Representative immunoblots from three independent experiments for CHOP, XBP-1, ATF-4 and lamin B are shown. Lamin B was used as the loading control for nuclear proteins. (**B**). Human THP-1-derived macrophages were treated with different concentrations of raltegravir (0, 5, 15, and 25 µM) in the absence or presence of lopinavir/ritonavir (LOPV/RITV, 25 µM) for 4 h. Representative immunoblots from three independent experiments for CHOP, XBP-1, ATF-4 and lamin B are shown.

**Figure 3:**
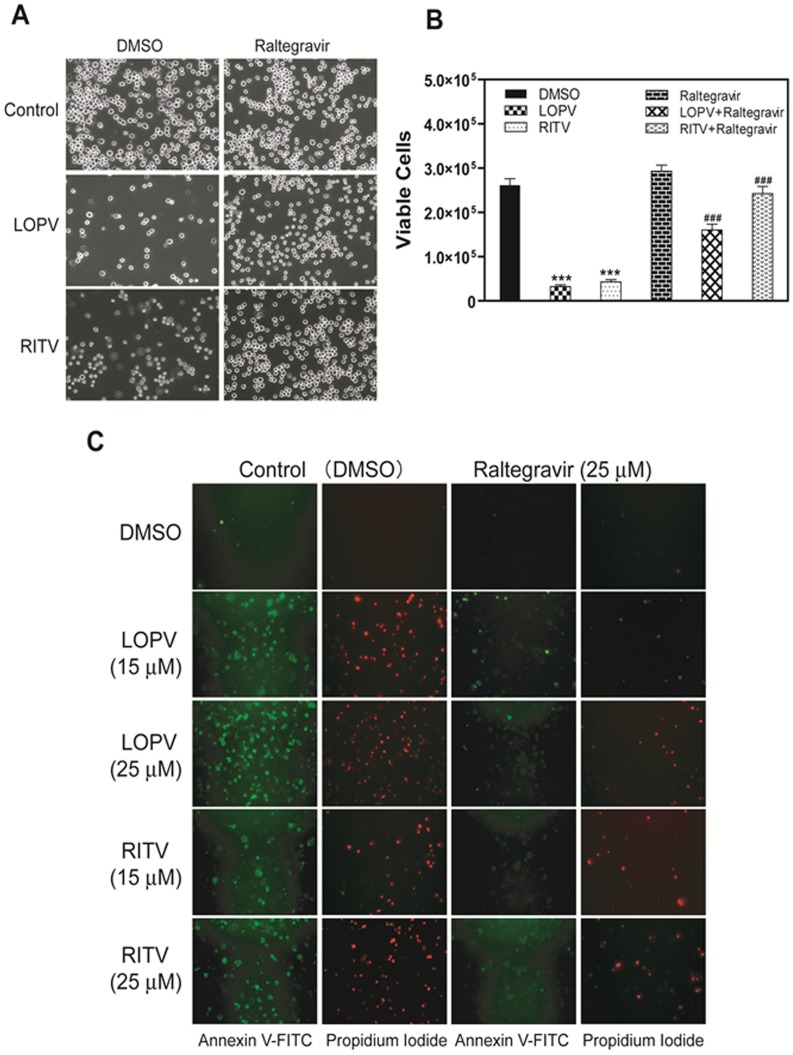
Effect of raltegravir on HIV PI-induced apoptosis in macrophages. Mouse J774A.1 cells were treated with lopinavir (LOPV, 25 µM) or ritonavir (RITV, 25 µM) with or without raltegravir (25 µM) for 24 h. At the end of treatment, the images of cells were taken using an Olympus microscope equipped with an image recorder. Cell viability was assessed with the TC10 cell counter (BioRad) and with the use of trypan blue. (**A**). Representative images of each treatment group. (**B**). Viable cell numbers from each treatment group. Values are mean ± S.E. of three independent experiments. ***, p<0.001, statistical significance relative to vehicle control. ###, p<0.001, statistical significance of HIV PI+raltegravir-treated group relative to HIV PI-treated group. (**C**). Human THP-1-derived macrophages were treated with different concentrations of HIV PIs (15 or 25 µM), lopinavir (LOPV), ritonavir (RITV) with or without raltegravir (25 µM) for 24 h. The apoptotic and necrotic cells were stained with Annexin V-FITC/Propidium Iodide and monitored as described in “Methods”. The representative images for each treatment are shown.

Our previous studies have shown that HIV PIs induce ER stress, activate the UPR, and promote apoptosis in macrophages. To determine whether raltegravir has a similar effect to HIV PIs on ER stress activation and induction of apoptosis in macrophages, we treated the J774A.1 cells and human THP-1-derived macrophages with raltegravir with or without ritonavir and lopinavir. The expression of UPR marker genes CHOP, XBP-1, and ATF-4 were determined by Western blot analysis. Thapsigargin, a known ER stress inducer, was used as a positive control. The apoptotic and necrotic cells were stained using Annexin V-FITC and Propidium Iodide and detected by fluorescence microscopy as described previously [5], [6]. As shown in Fig. 2, raltegravir did not activate the UPR, but significantly reduced HIV PI-induced expression of CHOP, XBP-1 and ATF-4 in mouse macrophages. In addition, treatment of the cells with raltegravir significantly prevented HIV PI-induced cell death (Fig. 3A and B). Similarly, raltegravir did not induce cell apoptosis, but it prevented HIV PI-induced cell apoptosis in human THP-1-derived macrophages (Fig. 3C).

### Effect of Raltegravir on HIV PI-induced Inflammatory Response in Macrophages

**Figure 4:**
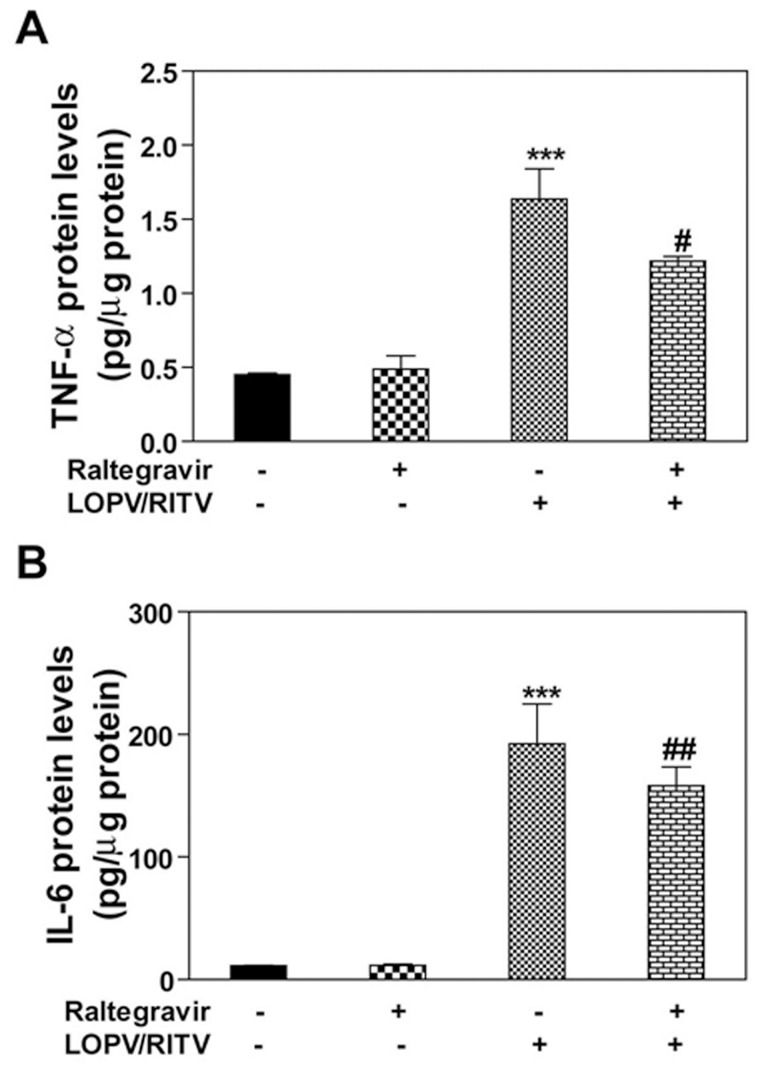
Effect of raltegravir on HIV PI-induced TNF-α and IL-6 expression in macrophages. Mouse J774A.1 cells were treated with HIV PIs (lopinavir/Ritonavir, 25 µM) with or without raltegravir (25 µM) or vehicle control (DMSO) for 24 h. At the end of treatment, the culture media and cells were collected separately. The amount of TNF-α and IL-6 released into the media were analyzed by ELISA and normalized with total protein amount of viable cells and expressed as pg/µg protein. (**A**). TNF-α,***, p<0.001, statistical significance relative to vehicle control. #, p<0.05, statistical significance of HIV PI+raltegravir-treated group relative to HIV PI-treated group. (**B**). IL-6,***, p<0.001, statistical significance relative to vehicle control. ##, p<0.01, statistical significance of HIV PI+raltegravir-treated group relative to HIV PI-treated group.

We have previously shown that HIV PIs induce the expression of proinflammatory cytokines (TNF-α and IL-6) in macrophages [4]. To examine whether raltegravir has a similar effect as HIV PIs on the expression of TNF-α and IL-6, J774A.1 cells were treated with raltegravir with or without lopinavir and ritonavir. The protein levels of TNF-α and IL-6 were determined by ELISA. As shown in Fig. 4, raltegravir did not induce the expression of TNF-α and IL-6, but significantly reduced HIV PI-induced expression of TNF-α and IL-6. Similar results were obtained in human THP-1 cell-derived macrophages (data not shown).

### Effect of Raltegravir on HIV PI-induced Foam Cell Formation in Macrophages

**Figure 5:**
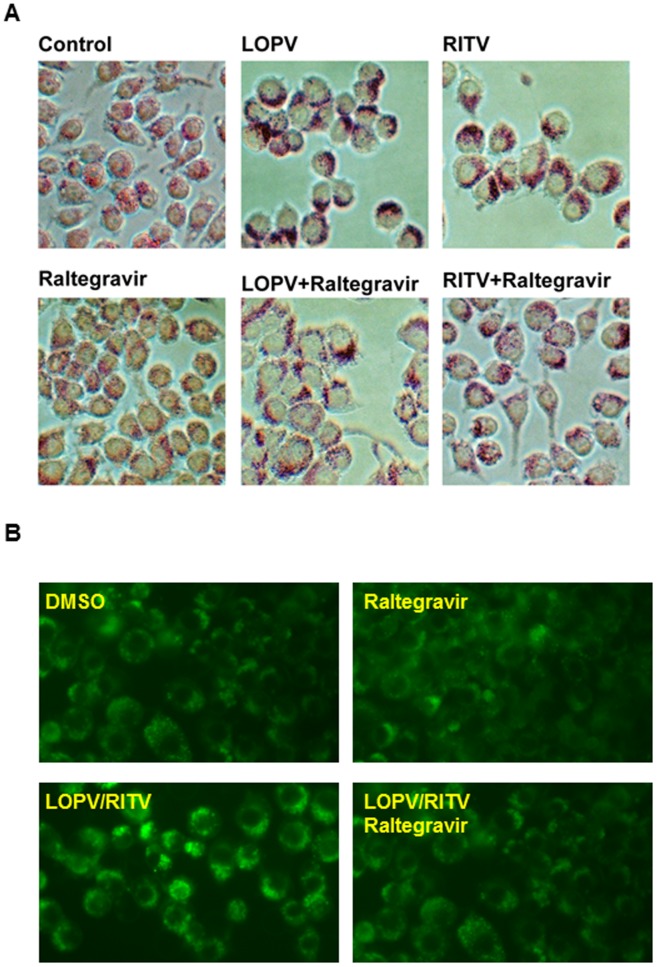
Effect of raltegravir on HIV PI-induced lipid accumulation in mouse J774A.1 macrophages. (**A**) J774A.1 cells were loaded with Acetylated-LDL (50 µg/ml) and treated with 25 µM of LOPV, RITV, or vehicle control with or without raltegravir (25 µM) for 24 h. The intracellular lipid was stained with Oil Red O as described under “Methods”. The images were taken with the use of an Olympus microscope equipped with an image recorder. The representative images are shown for each treatment group. (**B**). J774A.1 cells were loaded with oxidized-LDL (50 µg/ml) and treated with 25 µM of LOPV/RITV (4∶1), or vehicle control with or without raltegravir (25 µM) for 24 h. The intracellular lipid was stained with Nile Red as described under “Methods”. Images were obtained under a 40×objective using a FITC filter on a fluorescent microscope. The representative images are shown for each treatment group.

**Figure 6:**
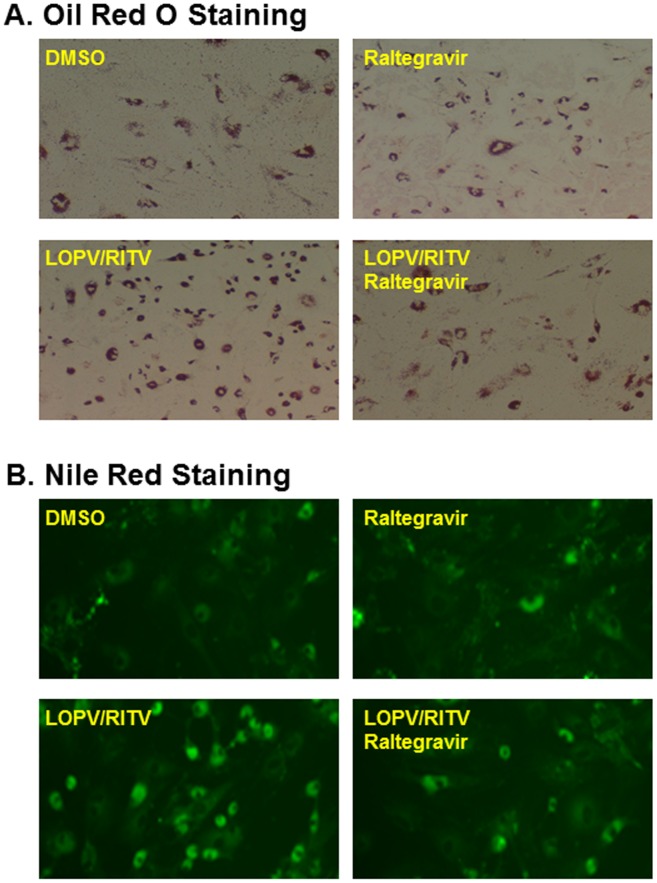
Effect of raltegravir on HIV PI-induced lipid accumulation in primary mouse peritoneal macrophages. (**A**). Primary mouse peritoneal macrophages were loaded with Acetylated-LDL (50 µg/ml) and treated with 25 µM of LOPV, RITV, or vehicle control with or without raltegravir (25 µM) for 24 h. The intracellular lipid was stained with Oil Red O or Nile Red and images were taken as described under “Methods”. (**A**). The representative Oil Red O staining images are shown for each treatment group. (**B**). The representative Nile Red staining images are shown for each treatment group.

**Figure 7:**
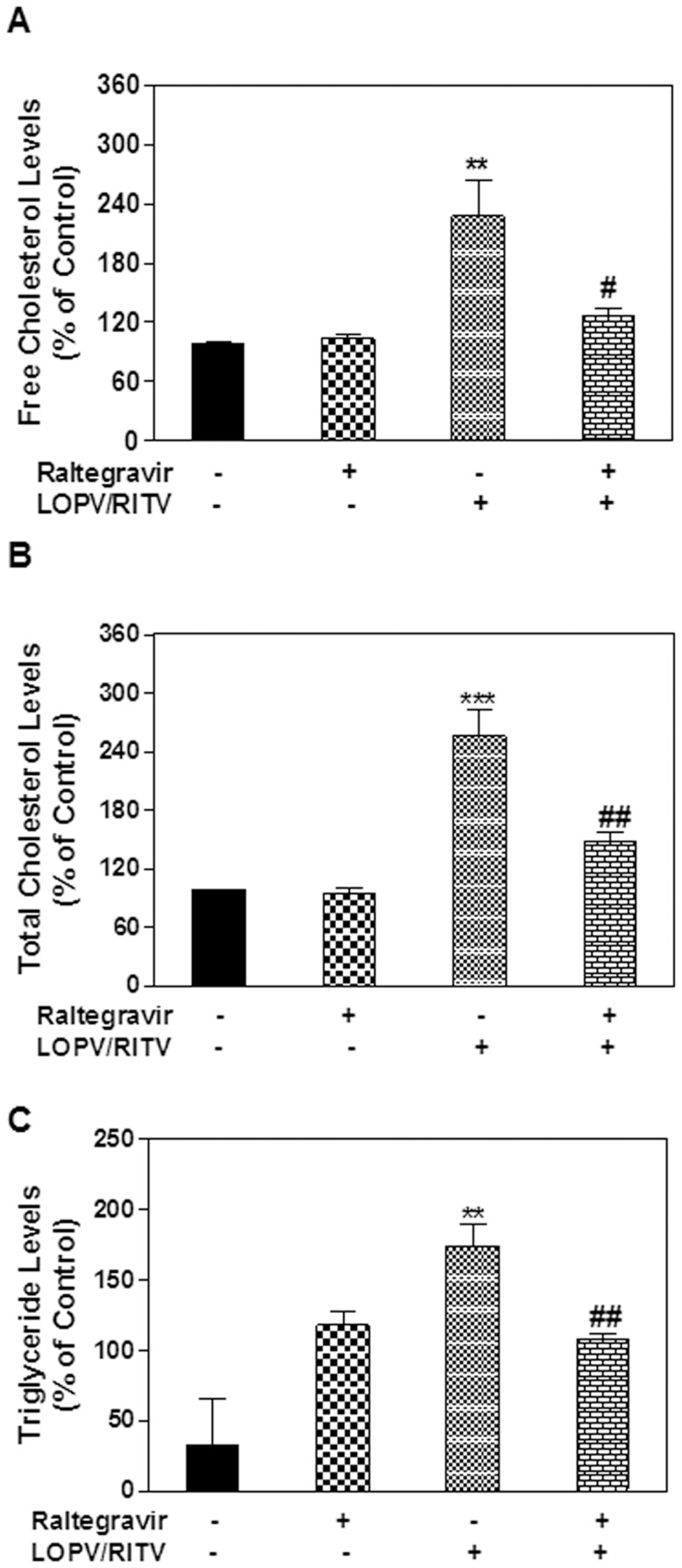
Effect of raltegravir on HIV PI-induced disruption of lipid homeostasis in macrophages. Mouse J774A.1 cells were treated with LOPV/RITV (15 µM) in the presence or absence of raltegravir (15 µM) for 24 h. Total cell lysates were used to measure the intracellular free cholesterol, total cholesterol, and triglyceride using Wako kits and normalized using total protein amount. The values are means ± S.E. of three independent experiments. **, p<0.01, statistical significance relative to vehicle control. #, p<0.05, statistical significance of LOPV/RITV+raltegravir-treated group relative to LOPV/RITV-treated group.

**Figure 8:**
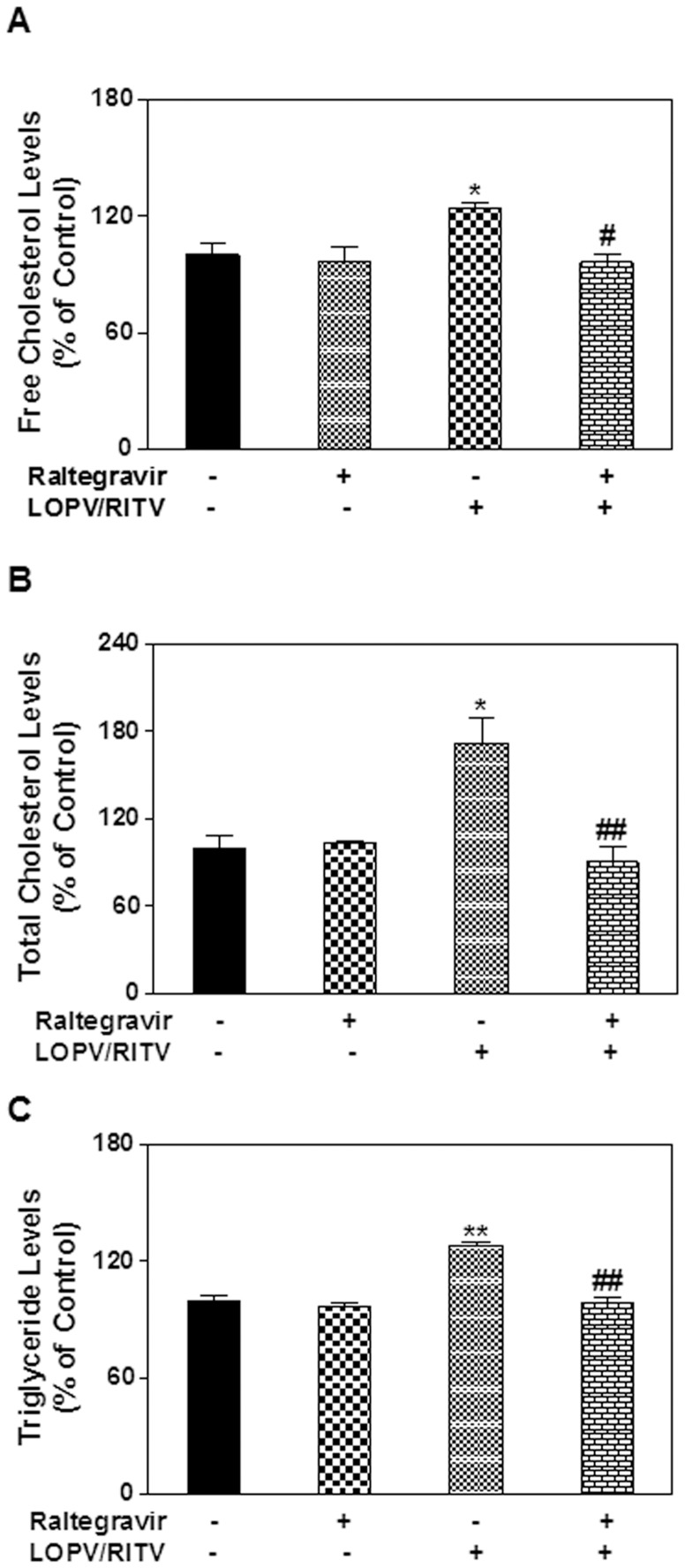
Effect of raltegravir on HIV PI-induced disruption of lipid homeostasis in primary mouse peritoneal macrophages. Mouse primary peritoneal macrophages were isolated as described in “Methods” and cultured for 48 h. Cells were treated with LOPV/RITV (25 µM) in the presence or absence of raltegravir (25 µM) for 24 h. Total cell lysates were used to measure the intracellular free cholesterol, total cholesterol, and triglyceride using Wako kits and normalized using total protein amount. The results are expressed as % of control. The values are means ± S.E. of three independent experiments. *, p<0.05; **, p<0.01, statistical significance relative to vehicle control. #, p<0.05; ##, p<0.01, statistical significance of LOPV/RITV+raltegravir-treated group relative to LOPV/RITV-treated group.

Maintenance of the cellular lipid homeostasis is crucial to various cellular functions. We have previously reported that HIV PIs induce intracellular free cholesterol accumulation and foam cell formation in macrophages [6]. To determine if the protective effect of raltegravir on HIV PI-induced UPR activation is correlated to its effect on lipid homeostasis, we examined the effect of HIV PIs on lipid accumulation in mouse primary Kupffer cells and peritoneal macrophages. As shown in online supplementary Fig. S1, HIV PIs increased intracellular lipid accumulation. Furthermore, our previous studies showed that HIV PIs induced foam cell formation in human and mouse macrophages [6]. We further examined whether raltegravir had any effect on lopinavir and ritonavir-induced foam formation in macrophages. Mouse J774A.1 cells were loaded with acetylated-LDL or oxidized-LDL and treated with HIV PIs with or without raltegravir for 24 h. The intracellular lipid was stained using Oil Red O or Nile Red as described previously [6], [18]. As shown in Fig. 5, raltegravir did not promote lipid accumulation in the presence of modified LDL, but reduced HIV PI-induced increase of lipid accumulation. Similar results were obtained in human THP-1 cells (data not shown). We further confirmed these observations in primary mouse peritoneal macrophages. As shown in Fig. 6, raltegravir also reduced lopinavir/ritonavir-induced uptake of oxidized-LDL and lipid accumulation in primary mouse peritoneal macrophages. Our previous studies have shown that ritonavir not only promoted the foam cell formation, but it also increased the intracellular free cholesterol accumulation in mouse macrophages [6]. In order to determine whether raltegravir has any effect on HIV PI-induced disruption of intracellular lipid homeostasis, we measured the intracellular free cholesterol, total cholesterol and triglyceride levels after the cells were treated with HIV PIs with or without raltegravir. As shown in Fig. 7, raltegravir significantly reduced lopinavir/ritonavir-induced increase of intracellular free cholesterol, total cholesterol and triglyceride in mouse J774A.1 macrophages. Similarly, lopinavir/ritonavir-induced increase of intracellular free cholesterol, total cholesterol and triglyceride was also inhibited by raltegravir in primary mouse peritoneal macrophages (Fig. 8).

### Effect of Raltegravir and HIV PIs on the Expression of Key Genes Involved in Regulation of Lipid Homeostasis in Macrophages

**Figure 9:**
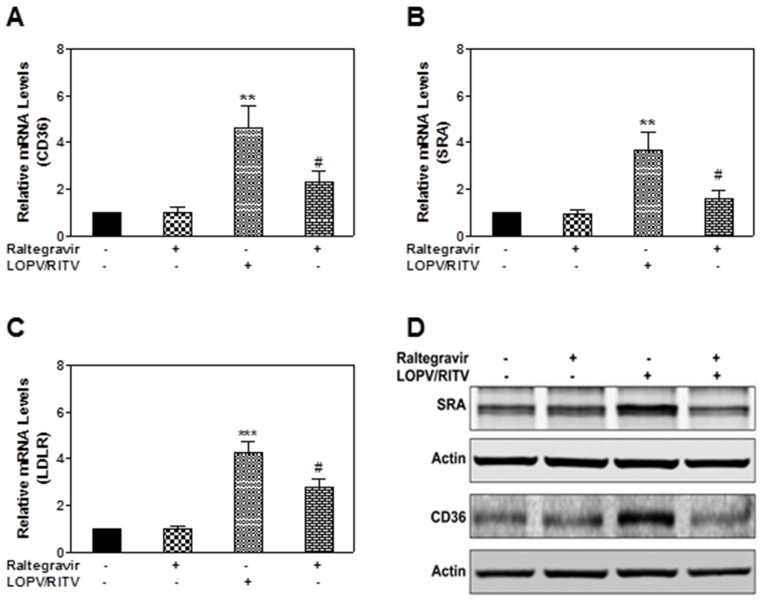
Effect of raltegravir and HIV PIs on the expression of key genes involved in lipid transport in macrophages. (**A–C**) Human THP-1-derived macrophages were treated with HIV PIs (lopinavir/Ritonavir, LOPV/RITV, 25 µM) with or without raltegravir (25 µM) for 24 h. The total cellular RNA was isolated and reverse transcribed. The relative mRNA levels of CD36, SRA, and LDLR were determined by real-time PCR as described in “Methods”. The values are means ± S.E. of three independent experiments. **, p<0.01, ***, p<0.001, statistical significance relative to vehicle control. #, p<0.05, statistical significance of HIV PI+raltegravir-treated group relative to HIV PI-treated group. (**D**) Human THP-1-derived macrophages were treated with HIV PIs (lopinavir/Ritonavir, LOPV/RITV, 25 µM) with or without raltegravir (25 µM) for 24 h. The total protein lysates were prepared and subjected to Western blot analysis. Representative immunoblots for CD36, SRA and β-actin are shown. β-actin was used as the loading control for total proteins.

In order to identify the mechanisms by which raltegravir prevents HIV PI-induced lipid accumulation in macrophages, we first examined the effect of raltegravir and HIV PIs on the expression of major surface receptors and transporters involved in lipid homeostasis including ABCA1, ABCG1, CD36, SRA, and LDLR in macrophages using real-time RT-PCR. As shown in Fig. 9, similar to our previous findings in mouse macrophages, HIV PIs (lopinavir/ritonavir) significantly increased the expression of CD36, SRA and LDLR in human THP-1-derived macrophages, which was inhibited by raltegravir. However, HIV PIs and raltegravir had no significant effect on the expression of ABCA1 and ABCG1 (data not shown).

### Effect of Raltegravir on HIV PI-induced Depletion of ER Calcium Stores in Macrophages

**Figure 10:**
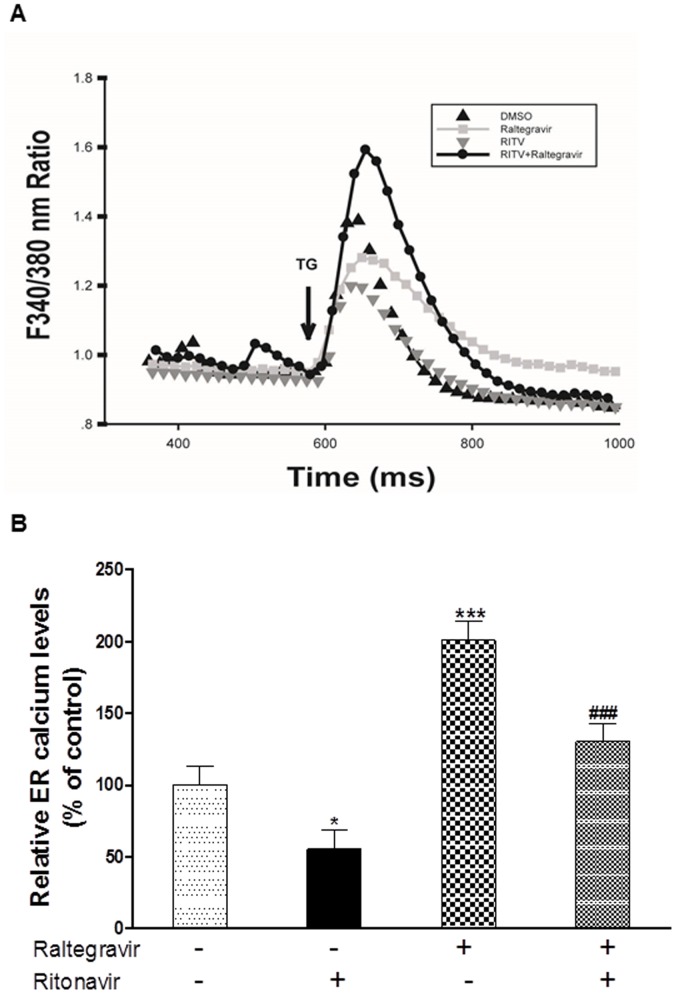
Effect of raltegravir on ritonavir-induced depletion of endoplasmic reticulum calcium stores in mouse macrophages. Mouse J774A.1 cells were treated with ritonavir (25 µM) with or without raltegravir (25 µM) for 24 h. (**A**) Representative tracings of the Fura-2 fluorescence ratio of 340∶380 nm in an individual macrophage for each treatment group before and after addition of 100 nM thapsigargin (TG) are shown. (**B**) Relative calcium content was calculated by total area under the curve for each treatment group and expressed as a percentage of vehicle control. Statistical significance relative to vehicle control, *, p<0.05; ***, p<0.001; Statistical significance relative to ritonavir group, ###,p<0.001.

Maintenance of ER calcium homeostasis is essential for many cellular functions. Disruption of ER calcium homeostasis is one of the major cellular mechanisms of the UPR activation. We have previously shown that HIV PI-induced ER stress activation is closely linked to its effect on ER calcium store depletion. In order to further determine whether raltegravir exerts its protective effect against HIV PI-induced ER stress through regulating ER calcium homeostasis in macrophages, we assessed the effect of raltegravir on ritonavir-induced ER calcium depletion in J774A.1 cells using the fluorescent calcium indicator fura-2/AM as described previously [6]. As shown in Fig. 10, raltegravir not only slightly increased the ER calcium store by itself, but also prevented ritonavir-induced reduction of ER calcium store.

### Effect of Raltegravir on HIV PI-induced Oxidative Stress in J774A.1 Cells

**Figure 11:**
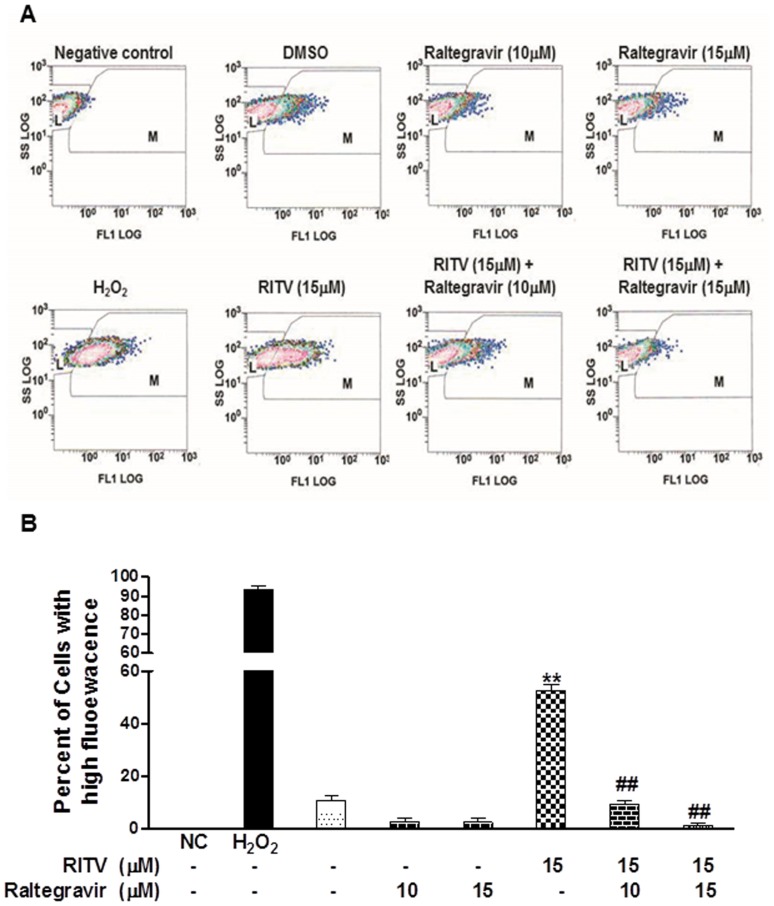
Effect of raltegravir on HIV PI-induced ROS production in macrophages. Mouse J774A.1 cells were treated with ritonavir (RITV, 15 µM) with or without raltegravir (10, or 15 µM) for 24 h, and then incubated with C-H_2_DCFDA-AM (2.5 µM) for 30 min (37°C). Cells without dye were used as negative control. Cells treated with H_2_O_2_ (300 µM) were used as positive control. Cells were collected, washed with PBS and analyzed by flow cytometry using a Beckman Coulter (EPICS XL). (**A**) The representative graphs for each treatment groups are shown. L and M represent the gates for no fluorescence and high fluorescence, respectively. (**B**) The percent of cells with high fluorescence for each treatment group is shown. Statistical significance relative to vehicle control, **, p<0.01; Statistical significance relative to ritonavir group, ##, p<0.01.

Numerous evidences indicate that ER stress activation is associated with induction of oxidative stress. We further examined the effect of raltegravir and HIV PIs on reactive oxygen species (ROS) production in J774A.1 cells by flow cytometry using C-H2-DCFDA as a fluorescent indicator. As shown in Fig. 11, ritonavir significantly increased ROS production, which was inhibited by raltegravir in a dose-dependent manner in macrophages. In order to exclude the possibility of HIV PI-induced increase of intracellular fluorescence due to the increase of dye up-take, we further examined the effect of HIV PIs and raltegravir on dye up-take using oxidized DCFDA. As shown in online supplementary Fig. S2, raltegravir had no effect on dye uptake, however, lopinavir/ritonavir significantly reduced dye up-take. These results further suggest that HIV PIs significantly increased cellular oxidative stress.

## Discussion

HIV PIs have been successfully used in HAART for HIV infection, which is the most effective treatment currently available. However, the benefit of HAART is compromised by HIV PI-induced metabolic syndrome and cardiovascular complications [2], [3], [7]. Until now, the underlying mechanisms behind serious HIV PI-associated metabolic abnormality and atherosclerosis in HIV-infected patients remain to be fully determined. Therapeutic and preventive strategies have yielded only limited success so far. Our previous studies and studies from other groups suggest that HIV PI-induced ER stress activation represents a critical cellular mechanism underlying HIV PI-induced inflammation, dyslipidemia, insulin resistance, and atherosclerosis [2], [5], [6], [19], [20]. It is well recognized that macrophages play a pivotal role in regulating inflammatory response and lipid metabolism.

Our recent studies indicated that raltegravir, the first agent in a new class of antiretrovirals, HIV integrase inhibitors, is not only less toxic, but also able to reduce HIV PI-induced dysregulation of lipid metabolism by inhibiting the ER stress response [15]. Based on the most recent guidelines for the use of antiretroviral agents in HIV-1-infected adults and adolescents released by the United States Department of Health and Human Services in February 2013, raltegravir has been listed as a component of the preferred regimens recommended for treatment-naive HIV-1-infected patients. Clinical studies have shown that raltegravir is well-tolerated with fewer side effects when compared to HIV PIs and reverse transcriptase inhibitors [21].

In the present study, we examined the effects of raltegravir on HIV PI-induced activation of ER stress, inflammation and dysregulation of lipid metabolism in cultured macrophages and mouse primary macrophages. The results demonstrated that raltegravir has less toxicity and could prevent HIV PI-induced ER stress activation, up-regulation of inflammatory cytokines, and foam cell formation (Figs. 4–6).

The role of ER stress is a rapidly emerging field of interest in the pathogenesis of various human diseases including liver diseases, metabolic syndromes, diabetes, inflammatory bowel diseases, and cardiovascular diseases [22]–[26]. Our previous studies demonstrated that activation of ER stress also plays a critical role in HIV PI-induced disruption of intestinal barrier integrity [27] and dysregulation of hepatic lipid metabolism [5]. Our recent studies have indicated that raltegravir could prevent HIV PI-induced apoptosis and disruption of lipid metabolism by inhibiting ER stress in rat primary hepatocytes and in liver [15]. In this study, we further showed that raltegravir also inhibited HIV PI-induced ER stress activation, apoptosis, and inflammatory response in macrophages.

The pathways of ER stress activation and lipid metabolism are woven together tightly. Lipid overloading in macrophages is a prerequisite for foam cell formation, which leads to the development of atherosclerotic lesions. Accumulation of intracellular lipid results when lipid influx exceeds lipid degradation and export. Activation of ER stress-mediated apoptosis will further increase the inflammatory response. Further analysis of the effect of raltegravir and HIV PIs on the expression of major surface receptors responsible for lipid uptake in macrophages suggested that raltegravir inhibited HIV PI-induced foam cell formation mainly by down-regulating the expression of CD36, SRA and LDLR (Fig. 9). It has been shown that atherosclerosis is a chronic inflammatory disease and activation of an inflammatory response is involved in all stages of atherosclerotic development [28]. We have previously shown that HIV PI-induced expression of inflammatory cytokines (TNF-α and IL-6) is coupled to the UPR and ERK signaling pathways [4], [6]. In this study, we found that raltegravir not only inhibited HIV PI-induced UPR activation, but also inhibited HIV PI-induced ERK activation (data not shown). Furthermore, raltegravir was able to reduce HIV PI-induced oxidative stress as indicated by reduction of ROS production in macrophages (Fig. 11). Activation of oxidative stress is closely associated with ER stress activation and has been linked to the pathogenesis of cardiovascular diseases and other inflammatory diseases [29]–[31]. We also showed that HIV PIs induce ROS production in cardiac myocytes [32].

Previous studies have shown that accumulation of intracellular free cholesterol induces ER stress by depletion of ER calcium store [33]. Our studies suggest that the raltegravir-mediated protective effect against HIV PI-induced dysregulation of lipid metabolism and activation of ER stress may act through the stabilization of the ER calcium content. The inhibition of CD36 and SRA expression may also prevent the uptake of modified lipids (oxidized LDL and Ac-LDL) by macrophages. Recent studies also suggest that ER stress and oxidative stress are also closely linked to autophagy, which has been recently identified as a key player in lipid metabolism [34]. Whether HIV PIs and raltegravir have any effect on autophagy activity remains to be further elucidated and is our current ongoing project.

In summary, the development of a HIV integrase inhibitor is a major step in the recent advance of anti-HIV therapy. Raltegravir not only increases the number of available agents to treat multidrug-resistant HIV infection with fewer side effects, but also provides a promising alternative in order to reduce the cardiovascular complications associated with commonly used anti-HIV agents. Our previous studies and the preliminary observations presented in this study suggest that HIV integrase inhibitors may be useful compounds not only for inhibiting multidrug-resistant HIV, but also for preventing HIV PI-associated metabolic complications. Our studies will provide important information for optimizing the combinational therapeutic regimen for HIV infection.

## Supporting Information

Figure S1 **Effect of raltegravir on HIV PI-induced lipid accumulation in primary mouse macrophages. A.** Primary mouse Kupffer cells were isolated from C57/BL6 wild type mice and cultured for two days and then treated with LOPV/RITV (15 µM) with or without raltegravir (15 µM) for 24 h. **B.** Primary mouse peritoneal macrophages were isolated from C57/BL6 wild type mice and cultured for three days and then treated with LOPV/RITV (15 µM) with or without raltegravir (15 µM) for 24 h. The intracellular lipid was stained with Oil Red O as described under “Methods”. The images were taken with the use of an Olympus microscope equipped with an image recorder. Representative images are shown for each treatment group.
(TIF)

Figure S2 **Effect of HIV PIs and raltegravir on fluorescent dye up-taking in mouse J774A.1 cells.** J774A.1 cells were loaded with the cell-permanent, oxidized form of the dye (DCFDA-AM, 5 µM) and treated with lopinavir/ritonavir (15 µM) with or without raltegravir (15 µM) for 4 h. The fluorescence intensity was measured using a 96-well plate reader. The values were means ± S.E. of three independent experiments. **, p<0.01, statistical significance relative to vehicle control.
(TIF)
