# Supplementary figures and images for: Reduction of the HIV Protease Inhibitor-Induced ER Stress and Inflammatory Response by Raltegravir in Macrophages
Source: PLoS One. 2014 Mar 13;9(3):e90856. doi: 10.1371/journal.pone.0090856 (PMC3953206; doi:10.1371/journal.pone.0090856)

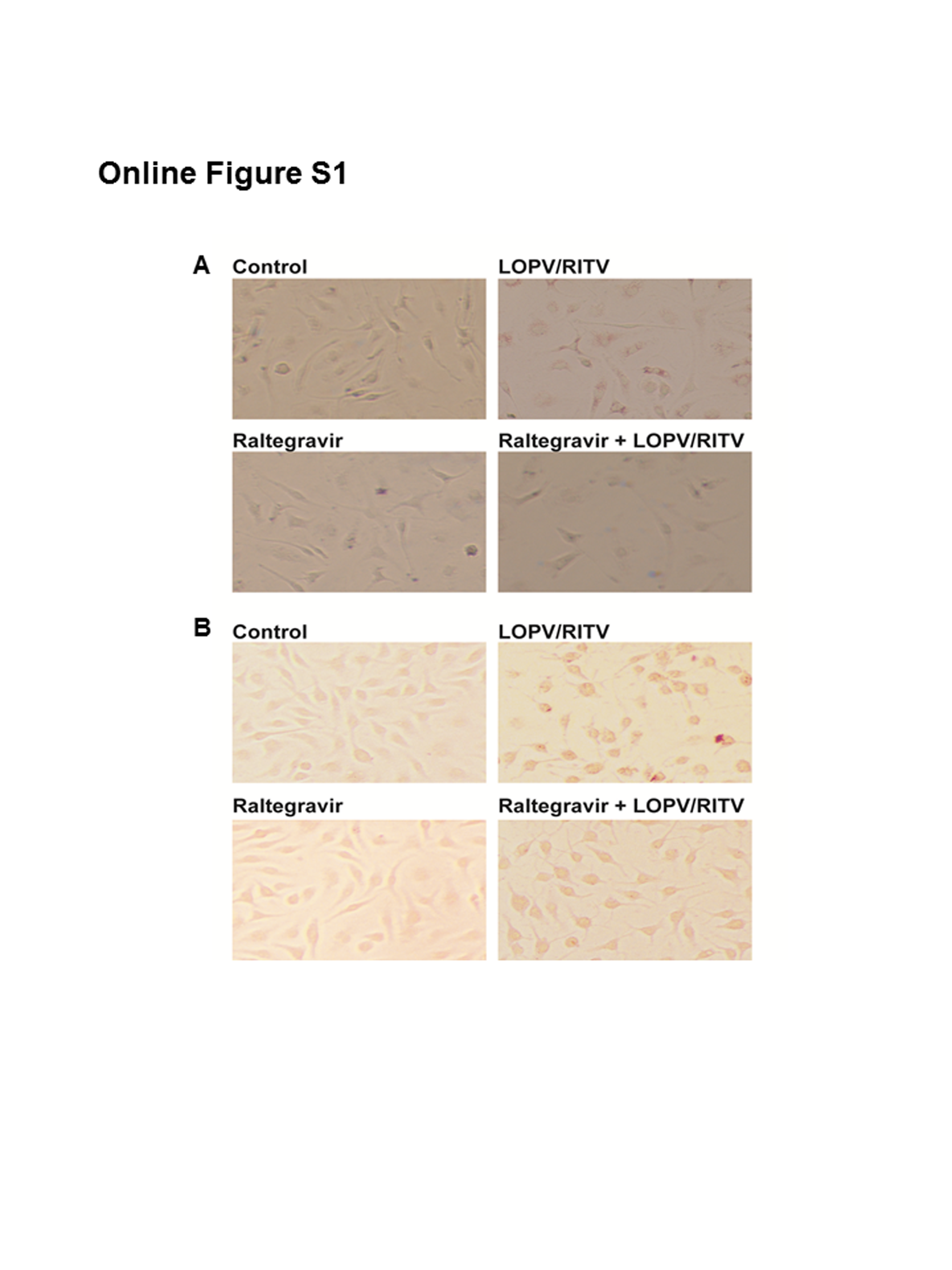

Supplement: Figure S1 — Effect of raltegravir on HIV PI-induced lipid accumulation in primary mouse macrophages. A. Primary mouse Kupffer cells were isolated from C57/BL6 wild type mice and cultured for two days and then treated with LOPV/RITV (15 µM) with or without raltegravir (15 µM) for 24 h. B. Primary mouse peritoneal macrophages were isolated from C57/BL6 wild type mice and cultured for three days and then treated with LOPV/RITV (15 µM) with or without raltegravir (15 µM) for 24 h. The intracellular lipid was stained with Oil Red O as described under “Methods”. The images were taken with the use of an Olympus microscope equipped with an image recorder. Representative images are shown for each treatment group. (TIF) [file pone.0090856.s001.tif]

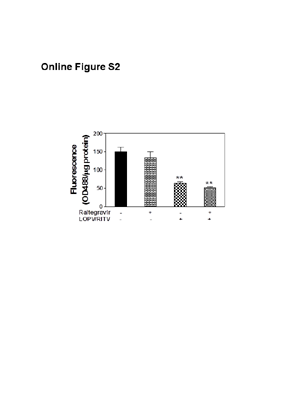

Supplement: Figure S2 — Effect of HIV PIs and raltegravir on fluorescent dye up-taking in mouse J774A.1 cells. J774A.1 cells were loaded with the cell-permanent, oxidized form of the dye (DCFDA-AM, 5 µM) and treated with lopinavir/ritonavir (15 µM) with or without raltegravir (15 µM) for 4 h. The fluorescence intensity was measured using a 96-well plate reader. The values were means ± S.E. of three independent experiments. **, p<0.01, statistical significance relative to vehicle control. (TIF) [file pone.0090856.s002.tif]
